# Colonization Screening Targeting Multidrug-Resistant Gram-Negative Pathogens Does Not Increase the Use of Carbapenems in Very Low Birth Weight Infants

**DOI:** 10.3389/fped.2020.00427

**Published:** 2020-08-06

**Authors:** Dominik Schöndorf, Arne Simon, Gudrun Wagenpfeil, Barbara Gärtner, Martina Geipel, Michael Zemlin, Marika Schöndorf, Sascha Meyer

**Affiliations:** ^1^General Pediatrics and Neonatology, University Hospital of Saarland, Homburg, Germany; ^2^Pediatric Oncology and Hematology, University Hospital of Saarland, Homburg, Germany; ^3^Theoretical Medicine, Institute for Medical Biometrics, Epidemiology and Medical Computer Sciences, University Hospital of Saarland, Homburg, Germany; ^4^Center for Infectious Diseases, Institute of Medical Microbiology and Hygiene, University Hospital of Saarland, Homburg, Germany

**Keywords:** colonization screening, multidrug-resistant Gram-negative pathogens, very low birth weight infants, carbapenem antibiotic consumption, antibiotic stewardship

## Abstract

Since 2012, a colonization screening (CoS) for multidrug-resistant Gram-negative bacteria (MRGN) in very low birth weight infants (VLWBI) was implemented in order to provide a basis for an effective empiric therapy of subsequent nosocomial infections (NI). According to antibiotic stewardship, carbapenems should be reserved for NI caused by MRGN or severe NI. We examined whether the CoS increased the first-line use of carbapenems. In this retrospective cohort analysis, we enrolled all VLBWI before (2009–2011) and after (2012–2014) the introduction of CoS (2012) at a tertiary university neonatal intensive care and neonatal intermediate care unit (NIMC) in Germany. Rectal swabs were used to detect MRGN colonization (on admission and weekly until discharge from the NIMC). The use of carbapenems was measured by days of therapy (DoT). To exclude the replacement of carbapenems by other antibiotics, antibiotic therapy for late-onset sepsis (LOS) was assessed by DoT and length of therapy (LoT). In 55/201 (27.4%) VLBWI, CoS detected MRGN colonization. Compared to the cohort prior to the introduction of CoS (*n* = 191), a significant decrease in LoT (*p* < 0.001) and total DoT (*p* < 0.001) was seen (*n* = 201). This was due to a significant decrease in LoT (*p* < 0.001) and total DoT (*p* < 0.001) in the birth weight category of 1,000–1,499 g. In these infants, DoT for carbapenems (*p* = 0.009) was significantly lower, possibly caused by a significant decline of LOS (25 episodes vs. 39 episodes, *p* = 0.025). Conversely, no significant differences in LoT and total DoT were seen in infants with a birth weight <500 g (*p* = 1.000; *p* = 0.758) and in infants weighing 500–999 g (*p* = 0.754; *p* = 0.794). DoT for carbapenems was not significantly different in the total cohort after the introduction of CoS (*p* = 0.341). Prolonged exposure to carbapenems (in terms of DoT) significantly postponed the first detection of MRGN colonization (*p* = 0.023). The introduction of CoS did not result in an increased use of carbapenems. Concomitant carbapenem treatment may reduce the sensitivity of CoS relying on rectal swabs.

## Introduction

Very low birth weight infants (VLBWI) have an increased risk for late onset sepsis (LOS) (1). In contrast to intrinsic risk factors (e.g., immature immune system and impaired skin and mucosal barriers) (1, 2), extrinsic risk factors (e.g., use of intravascular catheters, mechanical ventilation, etc.) can be successfully addressed by preventive bundles (1). Today the overall mortality of VLBWI is around 6–11%, with nosocomial infections (NI), e.g., LOS and necrotizing enterocolitis (NEC), being significant contributors (3). The attributable mortality of LOS is 9–15%, depending on clinical severity and causative pathogens (3). Survivors of LOS often suffer from neurologic sequelae (4). Nosocomial infections increase the costs during hospitalization and after discharge (5). Thus, prevention of NI aims at reducing mortality, short- and long-term morbidity, and healthcare expenditures (5, 6). Since the early signs and symptoms of LOS are often non-specific and LOS may progress to shock with multi-organ failure without timely and appropriate treatment, empiric antibiotic treatment is started at a low threshold in VLBWI with suspected LOS (1, 7, 8). VLWBI are mostly born by Cesarean section and subsequently colonized with bacterial pathogens from the surrounding neonatal intensive care unit (NICU) environment; colonization patterns differ between NICUs (2).

Late-onset sepsis caused by pathogens that colonize the gut and subsequently translocate into the bloodstream has been well-described (1, 9). Therefore, screening mucosal colonization by collecting nasal, pharyngeal, and rectal swabs may detect bacteria that are highly pathogenic (e.g., *Serratia marcescens*) (10) or multidrug resistant against different classes of antibiotics *in vitro* (11). However, the majority of NI are not caused by multidrug-resistant Gram-negative bacteria (MRGN) (12). Conversely, morbidity and mortality rates are higher in VLBWI suffering from LOS caused by MRGN, in particular when routinely used empiric antibiotic treatment is not effective against the causative pathogen (13). Thus, taking the results of the colonization screening (CoS) into account may increase the probability of choosing an effective antibiotic therapy (11). According to a recent survey in 80 NICUs in Germany, the great majority (94%) of neonatologists regularly recommend to adjust the antimicrobial treatment to the recent results of CoS (14).

A recent meta-analysis (1,984 neonates) demonstrated that 157 (7.9%) developed Gram-negative bloodstream infections compared with 85 of 3,583 (2.4%) non-colonized neonates (15). Some authors expressed the concern that the indiscriminate use of CoS targeting MRGN colonization in premature infants may increase the inappropriate use of carbapenems (16).

Most bacterial pathogens colonizing VLBWI are easily transmitted between patients (17), thus fostering the horizontal (nosocomial) transmission of MRGN between VLBWI on a NICU (2). In contrast to methicillin-sensitive and methicillin-resistant *Staphylococcus aureus* (18), no effective approach is available for MRGN decolonization (11).

Since 2012, the German Commission for Hospital Hygiene and Infection Prevention (KRINKO) recommends weekly CoS for MRGN in VLBWI treated in a NICU (19). To our knowledge, no other mandatory programs exist for CoS in the NICU in other countries (11), but up to 20% of NICUs in the United Kingdom do perform such a screening (20).

The use of 3rd-generation cephalosporins in NICUs has been linked to an increased risk of MRGN selection with a negative impact on the overall outcome (21). It is debated whether MRGN colonization increases the use of carbapenems as a first-line treatment when NI is suspected (8, 11, 16); such an increase would be in stark contrast to the concept of antibiotic stewardship (ABS) (7, 22). Carbapenems should be reserved for the targeted treatment of NI caused by MRGN or the empiric treatment of severe LOS and septic shock (11, 14). However, to avoid serious short- and long-term complications, neonatologists often decide to “err on the side of caution” (8).

Thus, the aim of this retrospective single-center cohort study was to investigate whether the introduction of CoS (as recommended by the KRINKO in 2012/2013) increased the use of carbapenems in VLBWI.

## Materials and Methods

### Setting and Study Period

This retrospective study was carried out at the tertiary University NICU and NIMC, General Pediatrics and Neonatology, University Hospital of Saarland, Homburg, Germany. All neonates with a birth weight less than 1.500 g (VLWBI), small for gestational age, and with intrauterine growth restriction admitted to the NICU for 3 years prior to the introduction of CoS (2009–2011) were compared to those VLBWI admitted in the 3 years after its introduction (2012–2014). Data were retrieved from our electronic in-hospital database as well as from hand-written medical charts for the complete hospital stay (admission to NICU until discharge from NIMC). Children surviving no longer than 24 h in the NICU as well as patients with incomplete data sets (missing information >10%) were excluded. The clinical risk index for babies (CRIB), as described by Cockburn et al. (23), was used to assess postnatal adaptation. We classified intraventricular hemorrhage (IVH) and bronchopulmonary dysplasia (BPD) as described by Papile et al. (24) and Jobe et al. (25), respectively.

### Microbiologic Testing: CoS, Blood, and Cerebrospinal Fluid Cultures

Prior to the introduction of CoS according to the KRINKO recommendation (19), a rectal swab was taken from all VLBWI on the day of admission (i.e., birth) to the NICU and cultured for bacterial growth routinely (CHROMagar™ ESBL, Paris, France; BD BBL™ MacConkey II Agar, BD Company, Franklin Lakes, NJ, USA). This approach was a common practice in Germany (19); its aims were to detect vertical transmission, prevent subsequent spread in the ward, and adjust the individual antimicrobial therapy of sepsis. In contrast to the latter mandatory CoS, further swabs to detect colonization only were taken at the discretion of the attending physician irrespective of a time interval. After CoS implementation, rectal swabs were collected on the day of admission/birth and repeated weekly until discharge from the NIMC. Not only MRGN but also highly pathogenic bacteria, e.g., *S. marcescens, Pseudomonas aeruginosa*, and *Enterobacter* spp., were reported by CoS in accordance with the KRINKO (26). For blood cultures, we used BD BACTEC™ Peds Plus™. This was also true for cerebrospinal fluid (CSF), which was only cultured if meningitis was suspected. Blood and CSF culture results were used to assess the number of culture-proven LOS. There was no written internal standard in our NICU/NIMC with regard to the collection of blood and CSF cultures in infants with suspected LOS.

### Definition of MRGN

In 2011, the KRINKO provided definitions for MRGN, mainly to facilitate infection prevention and control; Gram-negative pathogens with *in vitro* resistance against piperacillin and 3rd/4th-generation cephalosporins are defined as 3MRGN and as 4MRGN in case of *in vitro* resistance against carbapenems (27). The KRINKO recommendations on CoS in NICUs introduced a 3rd category (2MRGN NeoPed) for Gram-negative pathogens producing extended-spectrum beta-lactamases (ESBL) or AmpC-encoded resistance mechanisms (26). Since they are exclusively used in combination with other substances, the sensitivity to aminoglycosides is not part of the German MRGN definition (27); therefore, data on aminoglycoside resistance of the relevant pathogens, although reported in our hospital, are not shown in detail. After 2012, our hospital used the classification of bacterial resistance to antibiotics according to the KRINKO classification (26); the KRINKO definitions published in 2011 (27) and 2013 (26) were retrospectively used for all microbiological reports of isolates and their pattern of resistance before 2012. Time of first detection of MRGN was defined as the day when the particular specimen was cultured.

### Definition of NI and Ratios of Infection

To identify LOS, ventilator-associated pneumonia (VAP), NEC as well as device-associated LOS, we used the criteria of the German NEO-KISS surveillance system (6); in addition, NEC was classified as described by Bell et al. (28).

The days with use of vascular catheters [central venous catheters or umbilical catheters (CVC) and peripheral venous catheter (PVC)] as well as ventilatory support [endotracheal tube or non-invasive ventilation (NIV) including continuous positive airway pressure (CPAP)] were counted as device-days. If a CVC or PVC was used within 48 h before LOS developed, LOS was classified as CVC-associated or PVC-associated; if CVC and PVC were simultaneously present, LOS was recorded as CVC-associated. According to the above-mentioned criteria (6), the incidence density of LOS was calculated as the number of LOS per 1,000 patient-days; the device-associated rate of LOS was calculated as the number of these events per 1,000 device-days. Pneumonia was regarded as VAP if endotracheal mechanical ventilation or non-invasive ventilatory support (NIV and CPAP) had been provided within 48 h prior to the development of pneumonia.

As per the KRINKO recommendations, prospective NEO-KISS surveillance ends with a body weight of ≥1,800 g or when the infant is transferred from the NICU (6); in contrast, our surveillance period ended at discharge from the NIMC.

### Assessment and Choice of Antibiotic Use

The antibiotic treatment days for individual patients were evaluated in terms of DoT and LoT, regardless of the reason of therapy (postnatal, early-onset sepsis, LOS, perioperative, VAP, and NEC). While DoT accounts for every antibiotic drug used per day, LoT calculates the length of antibiotic treatment irrespective of how many substances are used per day. For example, treatment with ampicillin and gentamicin for 3 days results in a total of 6 DoT with a DoT of 3 for each of both antibiotic substances; the LoT is 3. Gentamicin was dosed according to gestational age (GA; e.g., every 48 h in infants with a GA less than 28 weeks), and only the number of days of gentamicin administration determined the corresponding DoT. The density of antibiotic treatment was defined as DoT per 1,000 patient-days with adjustment for the particular weight category (<500, 500–999, and 1,000–1,499 g) the patient belonged to.

Carbapenems and ciprofloxacin were defined as third-line antibiotics.

Since there was no written internal standard, the type of empiric treatment of suspected LOS was at the discretion of the attending physician; our standard antibiotic treatment, however, was the combination of aminopenicillin plus gentamicin as the first-line treatment and carbapenems as the second-line treatment, but modifications were possible at the discretion of the treating physician. The empiric treatment of LOS in MRGN-colonized VLBWI was at the discretion of the attending physician as well.

A “time-out” after 48 to 72 h of empiric treatment of suspected LOS was not established at our NICU.

When CVC-associated LOS was suspected, teicoplanin was used. In individual cases where the insertion of a CVC was very difficult (e.g., repeated venous puncture, prolonged time to insert), teicoplanin was given as a single shot to prevent associated LOS. According to the internal standard operation procedure, vancomycin was used as second-line treatment.

Since 2012, intravenous ampicillin–sulbactam (2:1) replaced mezlocillin due to a shortage in supply. Thus, no comparison between the cohort before and after the introduction of CoS was performed on the use of this first-line antibiotic.

### Ethical Issues

All data were retrieved from primary patients' files. We used only routine clinical and laboratory data and pseudo-anonymized all results before further analysis. Furthermore, this observational study represents an internal audit as a measure of quality assurance and therefore does not require neither informed consent from patients' legal representatives nor approval by an ethics committee (waiver from the Ethics Committee of the Medical Association in Saarland, issued Nov. 13^th^, 2014) (29).

### Statistical Analysis

We compared continuous variables between the cohort before and after the introduction of CoS by Mann–Whitney *U*-test; Kruskal–Wallis test was used to compare the DoT of the three categories of birth weight. For comparison of categorical variables, we used chi-square and Fisher's exact test. We analyzed the influence of LoT and DoT for carbapenems, ciprofloxacin, cephalosporins, and aminopenicillins on the time of MRGN's first detection by Cox regression; this analysis was limited to the cohort with implemented CoS because colonization was performed weekly only in that cohort. The variables that were significant in the univariate Cox regression analysis were tested multiple times (backward elimination; Wald). A *p*-value < 0.05 was considered as statistically significant. We used SPSS (RRID:SCR_002865) for Windows Version 26.0 (IBM Corp., Armonk, NY, USA) for statistical analysis. Tables and figures were performed using SPSS and Word Version 16 (Microsoft Corp., Redmond, WA, USA).

## Results

### Study Population

In total, we included 392 VLBWI comprising both periods of observation for final analysis; 12 patients not meeting the pre-specified criteria were excluded (Table 1). The cohort born after the introduction of CoS (*n* = 201) had a significantly lower median birth weight (1,060 vs. 1,120 g, *p* = 0.046) and a significantly longer length of stay in the hospital (68 vs. 61 days, *p* = 0.015) but was not treated significantly longer in the NICU (*p* = 0.383). Postnatal adaption (CRIB score) did not differ significantly (*p* = 0.240) between the two cohorts. IVH of grade II and III (*p* = 0.043) and BPD of any grade (*p* = 0.028) were observed significantly more often in the CoS cohort (Table 2). Six VLWBI died from sepsis (three from early-onset sepsis and three from LOS). Sepsis-related mortality was lower in the CoS cohort, but this difference did not reach a statistical significance (*p* = 0.232). In 55/201 (27.4%) VLBWI, CoS detected MRGN colonization (Table 3). Gram-negative bacterial isolates with resistance to gentamicin were not observed.

**Table 1:** Comparison of very low birth weight infants characteristics between the two cohorts before and after the introduction of colonization screening (CoS).

		**Before CoS (*n* = 191)**	**After CoS (*n* = 201)**	***p*-value**
Birth weight, g (median, IQR; min., max.)		1,120 (IQR 870–1,310; min. 380, max. 1,490)	1,060 (IQR 735–1,340; min. 320, max. 1,490)	0.046
Birth weight category (*n*)	<500 g	2	18	0.002
	500–999 g	73	75	
	1,000–1,499 g	116	108	
Gestational age, weeks (median, IQR; min., max.)		29 (IQR 27.14–31.14; min. 21.14; max. 36)	28.85 (IQR 26.43–30.71; min. 23.42; max. 37.28)	0.174
Small for gestation age (*n*)		20	21	0.055
Intrauterine growth restriction (*n*)		39	62	0.055
Cesarean section (*n*)		185 (96.9%)	195 (97%)	0.610
Female (*n*)		96 (50.3%)	90 (44.7%)	0.162
Clinical risk index for babies score (median, IQR; min., max.)		2 (IQR 1–5, min. 0, max. 15)	2 (IQR 1–6, min. 0, max. 17)	0.240
In-hospital stay, days (median, IQR; min., max.)	Neonatal intensive care	29 (IQR 14–43, min. 0, max. 138)	27 (IQR 14–50, min. 0, max. 168)	0.383
	Neonatal intermediate care	31 (IQR 23–44, min. 0, max. 83)	39 (IQR 27–49, min. 0, max. 124)	0.001

*IQR, interquartile range*.

**Table 2:** Comparison of morbidity and mortality between the two cohorts before and after the introduction of colonization screening (CoS).

		**Before CoS**	**After CoS**	***p-*value**
		**(*n* = 191)**	**(*n* = 201)**	
Intraventricular hemorrhage grades II and III		19 (10%)	34 (17%)	0.043
Neurosurgical procedure	Rickham reservoir	3 (1.6%)	6 (3.0%)	0.324
	Ventriculo-peritoneal shunt	10 (5.2%)	12 (6%)	
Periventricular leukomalacia		10 (5.7%)	5 (2.6%)	0.106
Bronchopulmonary dysplasia		29 (15.2%)	55 (27.4%)	0.028
Deaths (*n*)	Due to sepsis	5 (2.6%)	1 (0.5%)	0.232
	Due to other causes	13 (6.8%)	14 (7%)	

**Table 3:** Comparison of very low birth weight infants (VLBWI) with multidrug-resistant Gram-negative bacteria (MRGN) detected by rectal swabs on the day of admission before the introduction of colonization screening (CoS) and VLBWI with MRGN detected by CoS (i.e., rectal swabs collected on the day of admission and repeated weekly) after its introduction.

		**Before CoS (*n* = 191)**	**After CoS (*n* = 201)**
MRGN	All	36/191 (18.8%)	55/201 (27.4%)
	2MRGN	34/36 (94.4%)	49/55 (89.1%)
	3MRGN	2/36 (5.6%)	5/55 (9.1%)
	4MRGN	0	1/55 (1.8%)

*An infant colonized by 2MRGN and 3MRGN was classified as 3MRGN*.

### Incidence of LOS and Device-Associated LOS

Blood cultures were obtained in 61 and 59% of LOS episodes before and after the introduction of CoS, respectively (*p* = 0.916); they were positive in 32 and 29% of LOS episodes (*p* = 0.938), coagulase-negative *Staphylococcus* (CoNS) included. A total of 23% episodes of LOS were caused by CoNS. This ratio of culture-proven LOS did not differ in the subgroup of birth weight category of 1,000–1,499 g. The incidence and the incidence density of LOS and the rate of LOS associated with vascular catheters (CVC and PVC) were not significantly different after CoS introduction (*p* = 0.611 and *p* = 0.071, respectively) (Table 4). There was one episode of LOS with a positive CSF culture (*Enterococcus faecalis*) after intrauterine myelomeningocele surgery; in this case, the blood culture was negative.

**Table 4:** Comparison of nosocomial infections, use of devices, length of therapy (LoT), and total days of therapy (DoT) between the two cohorts before and after the introduction of colonization screening (CoS).

	**Before CoS (*****n*** **= 191)**			**After CoS (*****n*** **= 201)**			
	**Median**	**IQR**	**Total**	**Median**	**IQR**	**Total**	***p*-value**
In-hospital stay (days)	61	45–82	12,118	68	47–100	15,011	0.015
Stay on neonatal intensive care (days)	29	14–43	6,029	27	14–50	7,206	0.383
Stay on intermediate care (days)	31	23–44	6,006	39	27–49	7,619	0.001
Episodes of early-onset sepsis (EOS)	0	0	32	0	0	31	0.720
Episodes of late-onset sepsis (LOS)	0	0–1	77	0	0–1	95	0.948
Central venous catheter (CVC) (days)	19	14–23	3,706	20	15–27	4,529	0.079
CVC-associated LOS	0	0–1	68	0	0–1	70	0.611
Peripheral venous catheter (PVC) (days)	6	3–12	1,663	7	3–15	2,131	0.209
PVC-associated LOS	0	0	16	0	0	33	0.071
Rickham catheter (days)	0	0	571	0	0	1,050	0.376
VPS (days)	0	0	447	0	0	552	0.740
Endotracheal tube (days)	2	0–6	828	3	0–7	1,195	0.071
Continuous positive airway pressure (days)	2	0–6	1,148	5	1–21	2,656	<0.001
Total DoT	26	17–38	5,635	20	11–33	5,146	<0.001
LoT	19	15–24	3,820	14	8–23	3,531	<0.001

*Per definition, the total number of EOS corresponds with the number of affected infants. This is in contrast to LOS since some infants suffered from several episodes of LOS*.

*IQR, interquartile range; VPS, ventriculoperitoneal shunt; Total DoT, sum of DoT per antibiotic substance*.

### Nosocomial Infections Other Than LOS

Since VAP was only seen in four patients, its contribution on the use of antibiotics was not further analyzed; also, NEC was not analyzed further since only 10 cases of suspected NEC (Bell stage I) were detected in the two cohorts.

### Late-Onset Sepsis Caused by MRGN

Prior to the introduction of CoS, two VLBWI died from a LOS caused by 2MRGN *Enterobacter cloacae* firstly detected in blood cultures, but not in the rectal swabs prior to the onset of symptoms. Empiric treatment with mezlocillin plus gentamicin was switched to carbapenem when 2MRGN was identified, but it failed to control the infection. In no other VLWBI were the blood cultures MRGN-positive. There was no case of LOS caused by a pathogen primarily detected by CoS.

### Antibiotic Use

**Figure 1:**
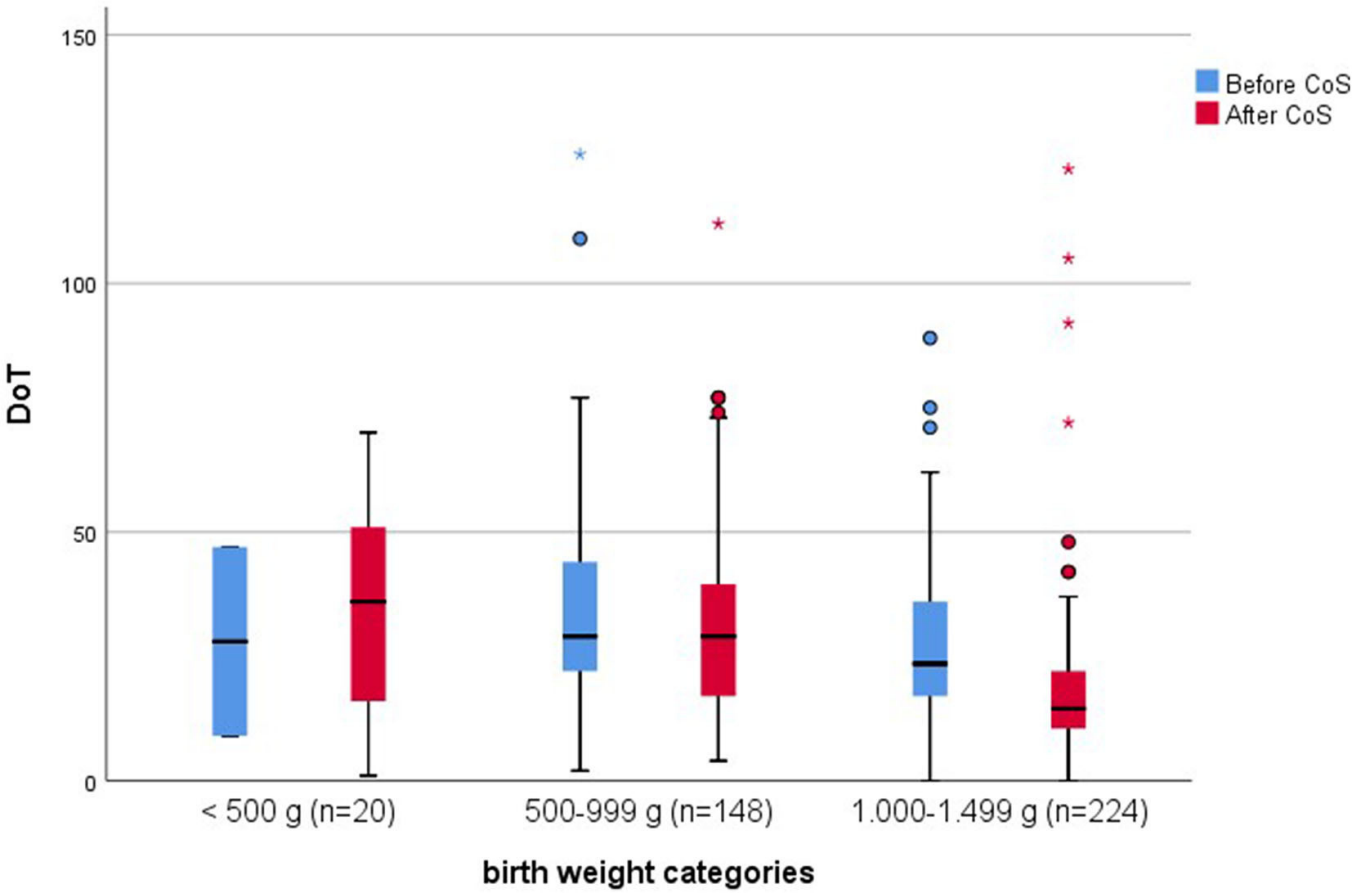
Comparison of total days of therapy (DoT; i.e., sum of DoT per antibiotic substance) between the two cohorts before and after the introduction of colonization screening, stratified by categories of birth weight.

After Cos was introduced, LoT was significantly shorter (*p* < 0.001), and total DoT was significantly lower (*p* < 0.001) (Table 4). The latter was caused by a significantly lower total DoT in the birth weight category of 1,000–1,499 g (*p* < 0.001) (Table 5; Figure 1); in particular, the DoT for carbapenems (*p* = 0.009) and teicoplanin (*p* = 0.040) was significantly lower (Table 6). This was due to a significantly lower prevalence of LOS (*p* = 0.025) and CVC-associated LOS (*p* = 0.048) in this particular category (Table 5).

**Table 5:** Comparison of of nosocomial infections, use of devices, length of therapy (LoT), and total days of therapy (DoT) between the two cohorts before and after the introduction of colonization screening (CoS), limited to the birth weight category of 1,000–1,499 g.

	**Before CoS (*****n*** **= 116)**			**After CoS (*****n*** **= 108)**			
	**Median**	**IQR**	**Total**	**Median**	**IQR**	**Total**	***p*-value**
In-hospital stay (days)	57	45–68	6,742	56	46–69	6,325	0.936
Stay on neonatal intensive care (days)	22	13–32	2,758	18	11–28	2,479	0.110
Stay on neonatal intermediate care (days)	32	26–44	3,901	37	25–46	3,713	0.410
Episodes of early-onset sepsis (EOS)	0	0	12	0	0	13	0.688
Episodes of late-onset sepsis (LOS)	0	0–1	39	0	0	25	0.025
Central venous catheter (CVC) (days)	18	14–21	2,121	17	14–22	2,154	0.948
CVC-associated LOS	0	0–1	37	0	0	24	0.048
Peripheral venous catheter (PVC) (days)	5	2–9	735	4	2–8	640	0.490
PVC-associated LOS	0	0	7	0	0	8	0.682
Rickham catheter (days)	0	0	104	0	0	342	0.154
VPS (days)	0	0	153	0	0	83	0.611
Endotracheal tube (days)	1	0–4	265	1	0–4	356	0.506
Continuous positive airway pressure (days)	1	0–3	316	3	1–6	632	0.004
Total DoT	24	17–36	3,130	15	11–22	2,097	<0.001
LoT	17	14–21	2,133	11	8–16	1,436	<0.001

*Per definition, the total number of EOS corresponds with the number of affected infants. This is in contrast to LOS since some infants suffered from several episodes of LOS*.

*VPS, ventriculoperitoneal shunt; Total DoT, sum of DoT per antibiotic substance*.

**Table 6:** Antibiotic use measured by days of therapy (DoT) and DoT per 1,000 patient-days (PD) between the two cohorts before and after the introduction of colonization screening (CoS), limited to the birth weight category of 1,000–1,499 g.

	**Before CoS (*****n*** **= 116)**		**After CoS (*****n*** **= 108)**		
	**PD 6742**		**PD 6325**		
	**DoT**	**DoT/1,000 PD**	**DoT**	**DoT/1,000 PD**	***p*-value**
Penicillin	0	0	8	1.3	0.300
Piperacillin	0	0	23	3.7	0.142
Piperacillin/Tazobactam	0	0	15	2.4	0.142
Cefuroxim	4	0.6	4	0.6	0.943
Cefotaxim	333	49.4	138	22.1	0.201
Ceftazidim	26	3.9	0	0	0.335
Fosfomycin	0	0	0	0	1.000
Gentamicin	347	51.5	318	51.0	0.555
Teicoplanin	250	37.1	165	45.5	0.040
Vancomycin	0	0	0	0	1.00
Carbapenems	309	45.8	160	25.7	0.009
Ciprofloxacin	35	5.2	31	5.0	0.208
Macrolides	357	53.0	212	34.0	0.192
Metronidazol	0	0	0	0	1.000
Linezolid	0	0	0	0	1.000

In contrast to the subgroup of birth weight category of 1,000–1,499 g, the DoT for carbapenems did not significantly vary between the two cohorts (*p* = 0.341) (Table 7). This was also true of LoT and total DoT (Figure 1), which did not differ significantly between infants with a birth weight <500 g (*p* = 1.000; *p* = 0.758) and between infants weighing 500–999 g (*p* = 0.754; *p* = 0.794) before and after the introduction of CoS. The LoT of a single LOS episode [median 7 days, interquartile range (IQR) 5–12] also did not differ significantly (*p* = 0,510) between the two cohorts.

**Table 7:** Antibiotic use measured by days of therapy (DoT) and DoT per 1,000 patient-days (PD) between the two cohorts before and after the introduction of colonization screening (CoS).

	**Before CoS (*****n*** **= 191)**		**After CoS (*****n*** **= 201)**		
	**PD 12.118**		**PD 15.011**		
	**DoT**	**DoT/1,000 PD**	**DoT**	**DoT/1,000 PD**	***p*-value**
Penicillin	12	1	19	1.3	0.967
Ampicillin/Sulbactam	11	0.9	2,008	133.8	–
Mezlocillin	2,398	197.9	136	9.1	–
Piperacillin	0	0	23	1.5	0.167
Piperacillin/Tazobactam	0	0	15	1	0.167
Cefuroxim	6	0.5	95	6.3	0.003
Cefotaxim	627	51.7	275	18.3	0.157
Ceftazidim	26	3	6	0.4	0.968
Fosfomycin	3	0.2	0	0	0.305
Gentamicin	620	51.2	634	42.2	0.991
Teicoplanin	524	43.2	692	46.1	0.847
Vancomycin	0	0	16	1.1	0.167
Carbapenems	756	62.4	654	43.6	0.341
Ciprofloxacin	71	5.9	32	2.1	0.046
Macrolides	543	44.8	561	37.4	0.993
Metronidazol	7	0.6	8	0.5	0.537
Linezolid	0	0	7	0.5	0.330

*The use of ampicillin/sulbactam and mezlocillin was not compared between the cohorts because ampicillin/sulbactam replaced mezlocillin since 2012*.

With regard to other last-resort antibiotics, the DoT for ciprofloxacin (*p* = 0.046) was significantly lower in the cohort after the introduction of CoS (Table 7). In total, seven VLBWI were treated with ciprofloxacin: three because of LOS without detection of a pathogen, two due to LOS caused by *P. aeruginosa*, one due to LOS caused by *Klebsiella* spp., and one LOS due to *Enterobacter* spp. Ciprofloxacin always was combined with other antibiotics. The DoT for cefuroxim was significantly higher after the CoS was implemented (*p* = 0.003) (Table 7).

### Time to First Detection of MRGN by CoS

After CoS introduction, the median time to the first detection of MRGN colonization was 43 days (interquartile range 26–86, min. 1, max. 133). In 23/55 (41.8%) VLBWI, a MRGN was detected for the first time during the stay in the NICU; in 32/55 (58.2%) cases, first detection occurred in the NIMC. None of the infants, in which the MRGN colonization was detected for the first time in the NIMC, developed LOS afterwards. On the other hand, in VLBWI with two or more episodes of LOS, any MRGN colonization was significantly later detected for the first time compared to those with no LOS (*p* = 0.021).

### Factors That Delayed the First Detection of MRGN by CoS

The DoT for aminopenicillins, cephalosporins, and ciprofloxacin (*p* = 0.218, *p* = 0.064, and *p* = 0.646, respectively) had no significant influence on the day of the first MRGN detection. The longer the LoT (*p* = 0.020) and the higher the DoT for carbapenems (*p* = 0.023), the significantly later the first detection by CoS. Given a DoT of 1 for carbapenems, the hazard ratio of MRGN's first detection was 0.951 (0.911–0.993); analogously, a DoT of 7 for carbapenems corresponded to a hazard ratio of 0.705. In multiple testing, the delay of MRGN detection by carbapenems was significant as well (*p* = 0.023).

## Discussion

Due to the morbidity, long-term sequelae, and attributable mortality, the prevention of LOS remains a major target in NICUs (4, 6). Since early and unspecific symptoms may rapidly progress to severe sepsis and septic shock with multi-organ failure, timely empiric antibiotic treatment is of utmost importance when LOS is suspected (1). Non-specific symptoms at an early clinical stage and missing laboratory parameters to definitely confirm or exclude LOS contribute to the use of broad-spectrum antibiotics (7).

Colonization with Gram-negative bacteria increases the risk of subsequent LOS (9), but it is impossible to predict with certainty that the pathogen causing LOS derives from the infant's colonizing microbiome (11, 30). When LOS is suspected, physicians must weigh the risk of higher morbidity and mortality due to inadequate empiric treatment against the best-practice attempts to administer broad-spectrum antibiotics judiciously (31). Unnecessary exposure to broad-spectrum antibiotics may select resistant pathogens, in particular if used for a prolonged period of time (1, 7). Most pathogens causing LOS are not MRGNs, but the mortality of LOS increases if MRGNs causing LOS are not covered by empirical treatment (11, 13, 31). Therefore, CoS aims at serving two important purposes: the choice of an effective empirical antibiotic therapy of NI in VLWBI with a history of MRGN colonization and the prevention of a horizontal transmission of MRGN and subsequent NI or outbreaks (12, 19). As shown in our study and by others (32, 33), 2MRGN NeoPed (e.g., ESBL-producing Gram-negative pathogens) are by far the most prevalent MRGNs in this high-risk population.

The treatment of LOS caused by an ESBL-producing Gram-negative pathogen requires the timely use of carbapenems (11). Since its first recommendation by the KRINKO, it has been debated whether CoS increases the unjustified/undue use of carbapenems as empiric treatment of LOS (8, 16). This may have a negative impact on antibiotic stewardship (7, 22). On the other hand, the DoT for carbapenems and LoT might not be substantially affected if carbapenems are used in MRGN-colonized infants as first-line empirical therapy but de-escalated to other antibiotics with a narrower spectrum of activity after an antibiotic “time-out” after 48 to 72 h of treatment (34); such an approach is part of an ABS program (34). Since no ABS program was implemented in our NICU/NIMC, a systematic de-escalation strategy was not followed.

Due to the different rates of antibiotic use, in particular concerning the use of third-line antibiotics (1), it is not possible to generalize our results. To our knowledge, this is the first study to demonstrate that CoS does not automatically increase the use of third-line antibiotics, in particular of carbapenems (8, 11, 16). Despite a significant lower birth weight without a significantly different rate of LOS, the cohort after the introduction of CoS had a significantly shorter LoT and lower DoT. This was due to the results in the birth weight category of 1,000–1,499 g, in which the prevalence of LOS was significantly lower after the CoS introduction. Subsequently, the DoT for carbapenems was significantly lower in this category. In the total cohort, CoS did not significantly alter the use of carbapenems; we can exclude that other antibiotic drugs (e.g., use of combinations) or the prolonged use of drugs replaced the carbapenems and therefore biased this result. We do not know why the rate of LOS declined in VBWI with a birth weight of 1,000–1,499 since the utilization rate of invasive devices was the same and no new NI prevention bundles were implemented in our NICU/NIMC. We can exclude that a more favorable risk profile of those VLBWI that were screened for MRGN was responsible for these results (35).

The median time point of first detection of MRGN by CoS was day 43 of life; in the majority of infants, this happened after the most critical period of neonatal intensive care. In this regard, MRGN colonization did not play a significant role in the risk calculation of the empiric use of carbapenems in our patient population during the early, most vulnerable phase of treatment.

Eighty-nine percent of MRGN detected by CoS were 2MRGN and only one 4MRGN isolate was detected. The longer the LoT and the higher the DoT for carbapenems, the first detection of MRGN appeared significantly later. Since carbapenems inhibit the growth of 2MRGN and 3MRGN, we hypothesize that concomitant treatment with a carbapenem may reduce the sensitivity of CoS. Lindner et al. reported a similar observation, confirming that concomitant antibiotic therapy may reduce the detection of pathogens in rectal swabs (36).

### Strengths and Limitations

Our study has several strengths: First, we analyzed all systemic antibiotics used during the hospital stay (NICU and NIMC); e.g., DoT was not lower due to the use of a broad-spectrum antibiotic instead of two or three antibiotics with a smaller spectrum of activity. This pitfall in the interpretation of antibiotic consumption data has been described by Kreitmeyr et al. (37). Second, we did not stop the further analysis of the prevalence of LOS, device-associated LOS, and antibiotic use when a weight of ≥1,800 had been gained by the individual VLWBI. This is in contrast to the common NEO-KISS surveillance system in Germany (6); we did so because CoS was not stopped at the end of the neonatal intensive care treatment but was continued until the patient was discharged for home from our NIMC. Interestingly, the majority of MRGN (58.2%) were detected on NIMC. The CoS at our NICU went beyond the KRINKO recommendations which only refer to VLBWI treated in the NICU (19). In our view, the analysis of MRGN prevalence in line with antibiotic use in NICU and NIMC provides internal quality control measures. This cannot be addressed solely by external surveillance (6). Third, neither changes in practice, i.e., preventive bundles, ABS, and standard operation procedures, nor changes in staffing were implemented, which may have biased our results.

Nevertheless, there are several limitations with regard to our study. First, since no ABS program was established, formal indications for antibiotic treatment, e.g., carbapenems, were never documented; we retrospectively interpreted their use in their clinical context. Second, we may underestimate the prevalence of VAP and its contribution to the use of third-line antibiotics. Third, we observed no definite episodes of NEC (Bell stages I and II), separating our tertiary NICU from many other NICUs where a NEC is a substantial contributor to NIs (38). The low incidence of VAP and NEC may result in the lower use of carbapenems but does not influence the analysis of CoS with regard to the use of carbapenems. Fourth, although CoS did not alter the low ratio of LOS episodes where a blood culture was not drawn before an antibiotic therapy (40.1 vs. 39%), MRGN as a cause of LOS could be underestimated.

## Conclusion

In this retrospective comparative cohort audit in a German tertiary NICU/NIMC, the introduction of CoS to identify VLBWI colonized with MRGN as recommended by the KRINKO did not increase the use of carbapenems as empirical treatment of suspected LOS. Aside from the birth weight category of 1,000–1,499 demonstrating a lower LOS prevalence of unknown cause, this was not due to a change in the rate of LOS or different risk factors in the cohorts compared. Since the first detection of MRGN by CoS mainly occurred after the critical phase of intensive care therapy, it did not influence the use of carbapenems. The use of carbapenems significantly delayed the first detection of MRGN; this suggests that the sensitivity of CoS for the detection of MRGN gut colonization may be reduced. The sensitivity of CoS for MRGN during concomitant treatment with carbapenems should be investigated in further studies, probably including culture-independent methods of detection (19).

## Data Availability Statement

The datasets generated for this study are available on request to the corresponding author.

## Ethics Statement

The studies involving human participants were reviewed and approved by Ethics Committee of Ärztekammer des Saarlandes. Written informed consent from the participants' legal guardian/next of kin was not required to participate in this study in accordance with the national legislation and the institutional requirements.

## Author Contributions

DS collected data, carried out data analysis, and drafted and revised the manuscript. MS collected data and carried out data analysis. SM and AS conceptualized the study and critically reviewed and revised the manuscript. GW supervised the statistical analysis and interpretation of the results. BG and MZ critically reviewed the manuscript. MG supervised the data collection. All authors contributed to the article and approved the submitted version.

## Conflict of Interest

AS is the second chair of the German Commission for Hospital Hygiene and Infection Prevention (KRINKO) affiliated at the Robert Koch Institute, Berlin. He coordinates the KRINKO working group on neonatal intensive care. Furthermore, he is the second chair of the German Society for Pediatric Infectious Diseases (DGPI) and coordinator of their working group on Antibiotic Stewardship. MZ coordinates the Recommendation “Bakterielle Infektionen bei Neugeborenen” (AWMF Registration Nr. 024/008). SM is chief investigator of the NeoVitaA Trial (DFG ME 3827/1-2). The remaining authors declare that the research was conducted in the absence of any commercial or financial relationships that could be construed as a potential conflict of interest.
